# Prognostic Value of Invasion, Markers of Proliferation, and Classification of Giant Pituitary Tumors, in a Georeferred Cohort in Brazil of 50 Patients, with a Long-Term Postoperative Follow-Up

**DOI:** 10.1155/2016/7964523

**Published:** 2016-08-18

**Authors:** Juliano Coelho de Oliveira Zakir, Luiz Augusto Casulari, José Wilson Corrêa Rosa, João Willy Corrêa Rosa, Paulo Andrade de Mello, Albino Verçosa de Magalhães, Luciana Ansaneli Naves

**Affiliations:** ^1^Department of Endocrinology, Faculty of Medicine, University of Brasilia, Brasilia, Brazil; ^2^Institute of Geosciences, University of Brasilia, Brasilia, Brazil; ^3^Department of Neurosurgery, Faculty of Medicine, University of Brasilia, Brasilia, Brazil; ^4^Department of Pathology, Faculty of Medicine, University of Brasilia, Brasilia, Brazil

## Abstract

Although some pituitary adenomas may have an aggressive behavior, the vast majority are benign. There are still controversies about predictive factors regarding the biological behavior of these particular tumors. This study evaluated potential markers of invasion and proliferation compared to current classification patterns and epidemiogeographical parameters. The study included 50 patients, operated on for tumors greater than 30 mm, with a mean postoperative follow-up of 15.2 ± 4.8 years. Pituitary magnetic resonance was used to evaluate regrowth, invasion, and extension to adjacent tissue. Three tissue biomarkers were analyzed: p53, Ki-67, and c-erbB2. Tumors were classified according to a combination of histological and radiological features, ranging from noninvasive and nonproliferative (grade 1A) to invasive-proliferative (grade 2B). Tumors grades 2A and 2B represented 42% and 52%, respectively. Ki-67 (*p* = 0.23) and c-erbB2 (*p* = 0.71) had no significant relation to tumor progression status. P53 (*p* = 0.003), parasellar invasion (*p* = 0.03), and classification, grade 2B (*p* = 0.01), were associated with worse clinical outcome. Parasellar invasion prevails as strong predictive factor of tumor recurrence. Severe suprasellar extension should be considered as invasion parameter and could impact prognosis. No environmental factors or geographical cluster were associated with tumor behavior.

## 1. Introduction

Pituitary adenomas are mostly benign and their first symptoms are related to hormonal hypersecretion or to hypopituitarism, when there is a compression of normal pituitary tissue [1, 2]. Some of these tumors can be associated with signs of infiltration, destruction, and invasion of neighboring tissues during their development, known as invasive pituitary adenomas. This term was first proposed by Jefferson [3, 4], who considered these adenomas local malignant tumors. However, there is still no clear definition of aggressiveness. Some authors suggest that they are often recurrent tumors, presenting a large volume, accelerated growth, invasion of neighboring structures, and resistance to multimodal (surgical, radiotherapy, and pharmacological) treatments [5–8].

In 2004, the World Health Organization (WHO) classified pituitary adenomas into typical adenomas, atypical adenomas, or carcinomas [8]. Unfortunately, this classification process does not establish a reliable relation with the clinical behavior or risk of carcinogenesis. Some typical pituitary adenomas show an aggressive phenotype, and some tumors considered as atypical pituitary adenomas may not be recurrent or resistant to pharmacological treatment. Even aggressive, nonmetastatic pituitary adenomas may show histopathological features similar to those of carcinomas [9]. These tumors should be diagnosed early, thus requiring close clinical and radiological monitoring. Intensive treatment protocols must be considered in these cases.

Recently, Trouillas et al. [10] proposed a prognostic clinicopathological classification, which considers both radiologic and histologic parameters and recommends a grading system as follows: 1A, noninvasive; 1B, noninvasive and proliferative; 2A, invasive and nonproliferative; 2B, invasive and proliferative; 3, metastatic. These authors [10] indicated a predictive prognostic value of the classification based on an 8-year follow-up. The role of clinical, radiological, and immunostaining of proliferation markers on tissue on clinical outcomes remains unclear, and the proposed categories of tumors need to be validated in longer-term studies of larger series. Furthermore, the influence of environmental factors on the outcome of these tumors has not yet been properly evaluated. The identification of the geographical distribution of patients, through the application of proper spatial distribution tools, may help describe some epidemiological aspects of the disease.

## 2. Objectives

The aim of this study is to analyze potential markers of aggressiveness and invasiveness, to compare them to current classification patterns, and to evaluate the geographical distribution of patients, correlating possible existing spatial trends. The spatial correlation applied seeks to identify the presence of disease clusters in the study region and to evaluate their prognostic impact on clinical outcomes after a long-term follow-up of a cohort of patients from the Brasilia area.

## 3. Material and Methods

This is a retrospective observational study of a nonrandomized, noncontrolled cohort. Data were gathered and individuals were recruited from February 2013 to December 2014 at the Neuroendocrinology Unit of the University Hospital, located in Brasilia, Brazil. From a preliminary review of around 500 medical records of patients with pituitary macroadenomas, 50 patients were selected according to the inclusion criteria. The study complied with the WMA Declaration of Helsinki and its amended versions on ethical principles for medical research involving human subjects and was approved by the Ethical Committee on Human Subject Research of the University of Brasilia.

### 3.1. Patients

The patients included in the study were those with a confirmed diagnosis of pituitary macroadenoma with signs of tumoral invasion and/or suprasellar extension who had undergone transsphenoidal neurosurgery and been submitted to immunohistochemical analysis of tissue for hormones (FSH, LH, TSH, GH, prolactin, and ACTH) and for proliferation markers Ki-67, p-53, and c-erbB2. All patients had periodical ambulatory follow-ups for at least 10 years during the 1984–2014 period, presenting at least one of the following criteria:

- initial tumor diameter larger than 30 mm;
- tumor regrowth of residual tissue following surgery;
- resistance to traditional treatment protocol.

The patient variables considered were address, age, gender, signs and symptoms at diagnosis, and comorbidities. Data concerning laboratory and radiological records during the long-term follow-up, focusing on residual tissue or tumor regrowth and signs of tumoral invasion and suprasellar extension, were analyzed. All patients signed a proper informed consent before participating in the study.

### 3.2. Radiological Evaluation

All image sections were reviewed, and the maximum pituitary lesion diameter values were determined before and after any relevant medical intervention was done. Cavernous sinus invasion was considered for cases in which tumor volume involved more than 2/3 of the internal carotid [11] or tumors grades 3 and 4 based on Knosp et al. [12] and Edal et al. [13] classifications, respectively. Sphenoidal sinus invasion was considered for cases in which there had been erosion of the sellar floor and/or tumor invasion of the sphenoid sinus on MRI (grades 1 and 2, according to Edal et al. [13]). Tumors with significant suprasellar extension (grade 4, Edal et al. [13]) that were causing obstructive hydrocephalus under close contact with the third ventricle and in the vicinity of the brain parenchyma tissue were also considered as invasive.

### 3.3. Immunohistochemistry Protocol

An immunohistochemistry analysis of the samples was previously performed at the Pathology Unit of the University Hospital of Brasilia, and the preliminary results were published [14, 15].

Specimens were fixed in 10% formalin and embedded in paraffin according to standard histological procedures. Hematoxylin and eosin staining was used in all sections. Immunohistochemical evaluation included hormonal and proliferation markers. Detailed information on the antibodies, clones, and dilutions used in the procedure had been already published [14, 15] (Table 1). Reactions were developed with diaminobenzidine (DAB) and counterstained with hematoxylin. The hormonal profile of pituitary tumors included GH, prolactin, LH, FSH, ACTH, and TSH. Proliferation markers Ki-67, p53, and c-erbB2 were obtained in a semiquantitative method. The results were considered positive for cases where p53 ≥ 3+ (immunoexpression from 25 to 50% of cells), Ki-67 ≥ 2+ (immunoexpression from 10 to 25% of cells), and c-erbB2 ≥ 2+ (positivity in more than 10% of cells) according to local protocols [14, 15] (Appendix B).

Immunohistochemistry was performed on paraffin block-embedded material stored at the archives of the Department of Pathology, University of Brasilia. The specimens were obtained during operative procedures intended to cure or limit the extension of the disorders, for which informed consent was obtained from all patients.

Specimens were fixed in 10% formalin and submitted to embedding in paraffin according to standard histological procedures. Hematoxylin and eosin staining was performed in all sections. Reactions were developed with diaminobenzidine (DAB) and counterstained with hematoxylin. Immunohistochemical evaluation (using Streptavidin-Biotin systems) included hormonal as well as proliferation markers. The immunostaining was performed in a semiquantitative method, based on a visual scale. The used antibodies, clones, and respective dilutions were previously validated and published by our research group [14, 15].

### 3.4. Clinicopathological Classification

Tumors were classified based on a combination of their histologic and radiological features. Adenomas were considered invasive when they presented growth to the parasellar region, to the sphenoid sinus, and to the suprasellar region, compression of the third ventricle, or the neighboring brain parenchyma. Proliferation was defined when the presence of at least two of the p53 ≥ 3+, Ki-67 ≥ 2+, or c-erbB2 ≥ 2+ required criteria were found. The grading system used included the following levels: 1A noninvasive; 1B noninvasive and proliferative; 2A invasive and nonproliferative; 2B invasive and proliferative; 3 metastatic [10].

### 3.5. Geographic Information Systems Analysis

Data treatment using geographic information systems (GIS) includes the processing of geographic data combined with satellite remote sensing imagery. They are used to create maps and to cross-correlate public health registries and other clinical records with environmental data. These data processing tools have recently enabled the use of GIS to evaluate the geographical patterns of the distribution of acromegaly in the central region of Brazil [16].

Brasilia is roughly located in the mid-western region of Brazil and University Hospital is the reference medical center for the treatment of pituitary tumors in the geographical region considered in this study. The population of Brasilia is comprised of individuals that have migrated from several remote regions of Brazil over the past 50 years, thus representing a multiracial sample.

In this study, clinical data were added to a database file structure created to provide adequate input for the chosen GIS software (ArcGIS version 9.3, ESRI, Redlands, CA). The residential addresses reported by the participants, selected according to diagnosis groups, were registered for spatial analysis. Geocoding parameterization from the Brazilian Portal Service was used to obtain coordinates of the home address of each patient. To ensure privacy protection, as recommended by the Health Insurance Portability and Accountability Act (HIPAA), IOM privacy regulations were observed in this study [17, 18].

The chosen scale of the maps was 1 : 300,000, and no geocodes (latitude and longitude) were shown in order to preserve individual confidentiality. The Jenks optimization method, which is a classification method often applied to GIS analysis, was used to determine the best distribution of data values into different classes. The applied method seeks to minimize variance reduction within each class and to maximize variance between the several classes into which the original data was divided [19, 20].

### 3.6. Statistical Analysis

The Kolmogorov-Smirnov test was applied to quantitative variables to assess the Gaussian distribution for all the groups considered (therapeutical, evolution, and sex groups). Data from this first analysis were separated into different groups showing Gaussian distribution. The results were expressed as mean ± standard deviation, and Student's *t*- or ANOVA tests were performed. The Tukey-Kamer method was used for multiple comparisons of all possible pairs of means, based on a student range distribution. For the qualitative analysis of variables, the results were expressed as frequency, and the Pearson's chi-squared or Fisher exact test was performed. The level of significance was *p* < 0.05. On tables larger than 2 × 2, in which associations were statistically significant, a Pearson residual analysis was done to determine which category had the highest effect on the association between the variables [21]. Pearson residual was statistically significant when falling out of interval [−1.96; +1.96]. Analyses were performed using the SAS 9.4 software (SAS Institute 2012, NC, USA).

## 4. Results 

The cohort comprised 50 patients, 24 men and 26 women, with mean age of 34.8 ± 16.4 years (total range 11–73 years) at the first endocrine evaluation, with no differences between genders (*p* = 0.1557). The mean postsurgical follow-up time was 15.2 ± 4.8 years.

The maximum tumor diameter average identified by diagnosis was 44.7 ± 13.6 mm, and macroadenomas > 40 mm were present in 68% of the patients. A total of 84% of all tumors presented signs of parasellar invasion (22% unilateral invasion, 62% bilateral invasion). Infrasellar invasion was observed in 80% of all cases. Suprasellar extension, of any degree, in a frequency of 98% of the cohort was observed. Of these, third ventricle and/or the brain parenchyma tissue (grade 4, according to Edal et al. [13]) was present in 64% of all cases (Table 2).

**Figure 1:**
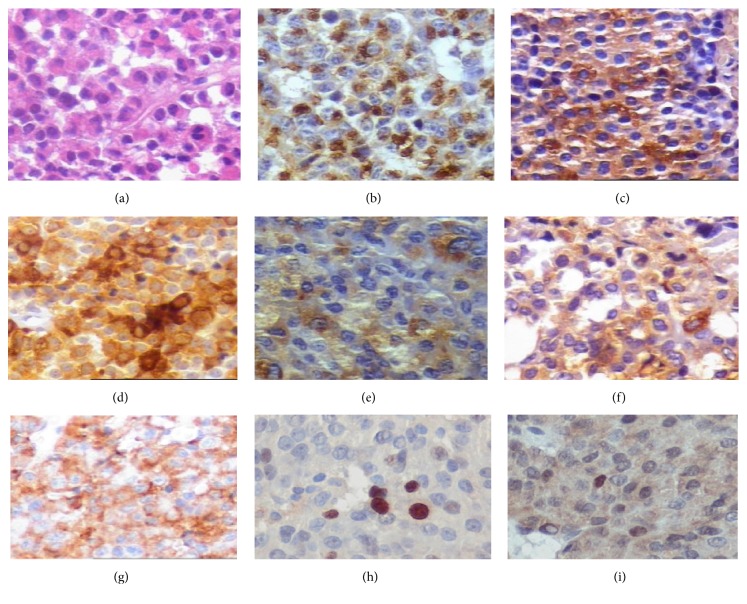
Immunohistochemical (IHC) analysis showing some pituitary adenomas: (a) hematoxylin-eosin; (b) prolactin; (c) GH; (d) LH; (e) FSH; (f) TSH; (g) c-erbB2; (h) Ki-67; (i) p53. The antibodies, dilutions, and reactivity scores were previously published [14, 15].

A review of the immunohistochemistry analysis (Figure 1) has shown that, of all cases, 36% were null cell adenomas, 28% were somatotropinomas, 16% were prolactinomas, 14% were gonadotropinomas, and 6% were silent corticotropinomas (Table 2). Most of the studied pituitary adenomas were sporadic, while 8% were considered as familial isolated pituitary adenomas (FIPA), with equal gender prevalence.

The observed frequency of immunoexpression of proliferation markers was 60% for p53 (≥3+), 33% for Ki-67 (≥2+), and 67% for c-erbB2 (≥2+). Tumors with immunoexpression of at least 2 markers in high proliferation index were observed in 54% of the cohort and were considered as proliferative adenomas.

**Figure 2:**
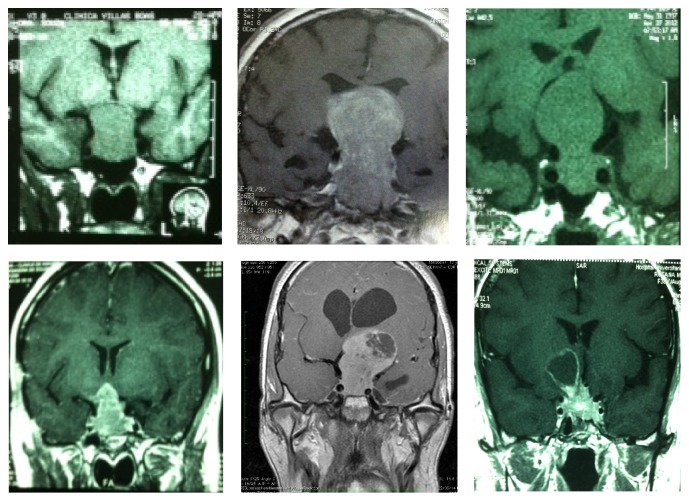
Radiological evaluation of aggressive pituitary tumors.

All radiological evaluation was reviewed, and images from diagnosis and during all follow-up period were considered. It was observed that 94% of the tumors were considered anatomically invasive by diagnosis (Figure 2). According to the clinicopathological classification, an association between anatomic and pathological classes revealed that 4% were noninvasive and nonproliferative tumors (grade 1A), and 2% were noninvasive and proliferative (grade 1B). Furthermore, 42% of the tumors from the total sample were invasive and nonproliferative (grade 2A), while 52% were invasive and proliferative tumors (grade 2B). No metastatic tumors were observed.

### 4.1. Relation of Proliferative Tissue Markers and Clinical Aspects

The intensity of immunohistochemical expression of tumor proliferation (Ki-67 and c-erbB2) and tumor suppressor markers (p53) and their relation to clinical and radiological aspects are listed in Table 3. Younger patients presented stronger immunostaining for p53 (>3+) and Ki-67 (>2+) (*p* = 0.021 and *p* < 0.001, resp.). No significant association could be observed between c-erbB2 expression and age at diagnosis.

Radiological aspects such as tumor size, infra- or parasellar invasion, and suprasellar extension were compared to tissue immunoexpression of proliferation markers (Table 3). Maximal tumor diameter was associated with stronger immunostaining for Ki-67 (*p* = 0.009), but no significant association was found for p53 (*p* = 0.062) and c-erbB2 (*p* = 0.937). Parasellar invasion was present in 94% of cases; however, invasion was not associated with proliferative markers.

Suprasellar extension in any degree was observed in all patients, except one, so, it was not possible to compare the groups for presence or absence of suprasellar extension. Although statistical analysis was done to test the impact of intensity of immunostaining for proliferation markers into the group of patients that presented suprasellar extension, no association was found. Extension to third ventricle was present in 64% of tumors and was related to p53 immunostaining (*p* = 0.013) but was not associated with the immunoexpression of Ki-67 and c-erbB2.

Regarding the hormonal profile, a strong expression of Ki-67 was more frequent in prolactinomas (*p* = 0.038). Immunoexpression of p53 was found in 88% of prolactinomas and c-erbB2 was found in 83% of null cell adenomas and 71% of gonadotropinomas.

### 4.2. Therapeutic Modalities

Patients from our cohort were treated by surgery only or by combined interventions, such as surgery followed by medical treatment or radiotherapy. The main treatment modality was surgery associated with medical treatment, observed in 40% of patients. Younger patients were submitted to multimodal treatments, including surgery and postoperative radiotherapy combined with medical treatment (*p* = 0.008).

There is a significant association between signs of infrasellar invasion and the applied therapeutical modality (*p* = 0.021). Patients that did not present infrasellar invasion were more frequently submitted to surgery alone (Pearson residual = +2), while those who showed infrasellar invasion were more commonly submitted to the combination of surgery and medical treatment (Pearson residual = +2) (Table 4).

A significant positive association was observed between hormone secretion and the applied therapeutic modality (*p* = 0.0186). Patients diagnosed with GH and PRL producing tumors (Pearson residual = +2) were mostly treated by surgery associated with drug treatment. Patients with null cell adenomas were more often treated with surgery followed by radiotherapy (Pearson residual = +2) (Table 5).

Tumors presenting a Ki-67 low proliferation index (<2+) were more often submitted to surgery alone and rarely to surgery followed by radiotherapy. Patients with adenomas presenting high Ki-67 immunoexpression had, as prevailing therapeutical intervention, surgery combined with drug therapy (*p* = 0.0110; Pearson residual = +2). There was no significant statistical association between the p53 and c-erbB2 tumor markers and the applied therapeutical modalities.

The most frequent adenomas were somatotropinomas (28%) and null cell adenomas (36%) (Table 5). The hormonal profile had impact on the choice of applied therapeutical strategies (*p* = 0.0186). Most of patients were from clinicopathological classes 2A and 2B but presence or absence of histological signs of proliferation did not change the applied treatments. It is worth mentioning that 43% of invasive adenomas were treated by surgery followed by complementary medical treatment.

### 4.3. Prognostic Impact

The impact of clinical and radiological aspects at diagnosis on clinical outcomes is expressed on Table 6. A greater rate of tumor regrowth/recurrence was observed among women (65%) and a higher prevalence of tumoral volumetric stability was seen among men (62%). However, there was no significant association between tumor behavior and gender (*p* = 0.3990), initial tumor diameter (*p* = 0.0524), or age at diagnosis (*p* = 0.4197) (Table 6).

Absence of parasellar invasion was associated with a higher frequency of tumor stability after treatment (*p* = 0.0389; Pearson residual = +3). However, parasellar invasion was not related to outcomes such as tumor regrowth/recurrence and cure/reduction. Infrasellar invasion and suprasellar extension were not considered as good prognostic markers of clinical outcome. Nevertheless, there was a tendency to associate absence of extension to third ventricle to a greater chance of tumor stability after treatment (Table 6).

By comparing the hormonal profile and the clinical outcomes, it was seen that 45% of somatotropinomas showed reduction of tumor volume or cure. However, there was no significant statistical relation between hormone secretion and prognosis of tumor groups (*p* = 0.2193).

The role of proliferative markers on clinical behavior was tested. The residual Pearson test was performed to identify which differences could lead to potentially rejecting the null hypothesis and highlight impacting variables. Preliminary data suggested that adenomas with a strong expression of p53 (≥3+) were associated with regrowth/recurrence (Pearson residual = +2); however, 67% of tumors with immunoreactivity for p53 were reduced or cured (Pearson residual = +1).

The low expression of p53 was related to tumor stability (Pearson residual = +3) (*p* = 0.0035), but not to tumor cure or reduction (Table 6). No significant differences were found concerning the expression of Ki-67 and c-erbB2 and clinical outcome.

With regard to anatomical classification, no statistical difference was observed in clinical outcome when comparing invasive and noninvasive adenomas. Nonproliferative tumors had a tendency to maintain volumetric stability (Pearson residual = +3; *p* = 0.0145), regardless of these tumors being invasive (grade 2A) or not (grade 1A) (Pearson residual = +2; *p* = 0.0127). However, proliferative tumors showed higher regrowth/recurrence rates (Pearson residual = +2; *p* = 0.0145), especially those classified as invasive and proliferative (grade 2B) (Pearson residual = +2; *p* = 0.0127) (Table 7).

Therapeutic interventions influenced clinical outcomes (*p* = 0.0272). Tumor stability was statistically associated with surgery followed by radiotherapy (Pearson residual = +2). Moreover, tumor cure/reduction was significantly achieved when surgery was associated with medical treatment (Pearson residual = +3).

Some tumors presented aggressive behavior despite multimodal treatment, and even when treated by surgery, radiotherapy combined with drug treatment presented tumor regrowth/recurrence (Pearson residual = +2). In these cases, multiple therapeutic interventions were not able to change the prognosis of the disease.

## 5. Application of Geographic Information Systems (GIS)

### 5.1. Spatial Analysis of Patient Cohort

**Figure 3:**
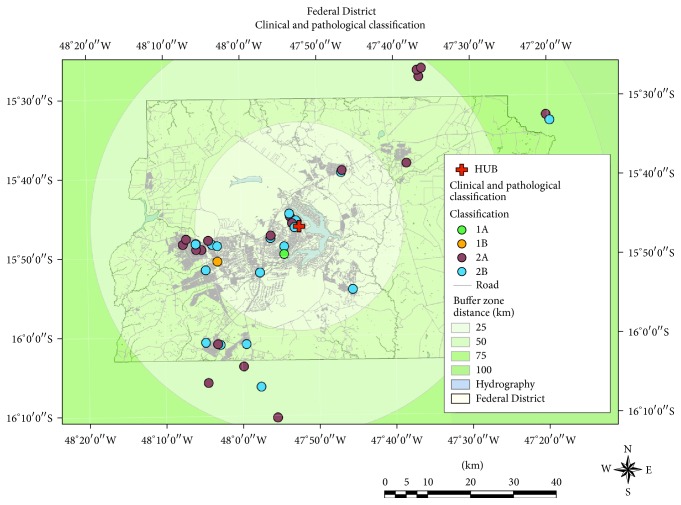
Georeferencing of patients with aggressive or invasive tumors. These include the following information: patients' home distance to Referral Medical Center (red cross). Buffer distances are represented in four zones, ranging from 25 to more than 100 km. Patients' clusters are expressed in different colors according to their geographic location (used GIS software: ArcGIS version 9.3, ESRI, Redlands, CA). Map scale 1 : 300,000.

Regarding the geographic analysis, the Federal District was represented in buffers corresponding to the radial distance to the medical care unit. Four zones were segmented, each at a distance of 25 km from the Referral Medical Center. The patients were grouped according to their clinicopathological classification, and the groups of patients living less than 25 km from the Referral Medical Center (RMC) were distributed as follows: 1A, 4.76%; 1B, 4.76%; 2A, 33.3%; 2B, 57.14%. Patients living 26–50 km away from the RMC were divided as follows: 2A, 66.6%; 2B, 33.3%. Patients living 51–75 km away from the RMC were grouped as follows: 2A, 50%; 2B, 50% (Figure 3).

**Figure 4:**
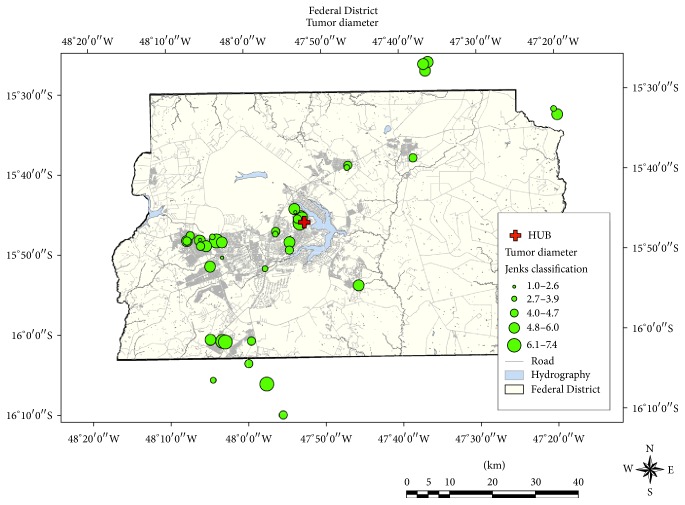
Geographic distribution of natural breaks classification results of tumor size, using Jenks classification.

The Jenks natural breaks classification method showed that 60% of patients had maximal tumor diameter ranging from 40 to 60 mm, 12% had tumors measuring from 61 to 70 mm, and 28% of all patients had tumors < 40 mm but presented invasion criteria (Figure 4). No unexpected geographically distributed clusters were found, and there was no resulting association between tumor grade and distance to the hospital where the patients were treated.

## 6. Discussion

This retrospective study compared the clinical and tissular aspects, their impact on tumor stability, regrowth or cure, and their relation to chosen therapeutic interventions. The identification of aggressive adenomas is a challenge, and the study of expression of factors that control cell proliferation (Ki-67, c-erbB2, and p53) and radiological signs of invasion may help to identify adverse prognostic factors [22].

The estimated prevalence of WHO atypical pituitary adenomas [8] varies from 2.7 to 15% [23, 24]. These tumors may have aggressive behavior and should be identified and treated earlier. Nevertheless, given the important aspects of this type of adenomas, there are no current standard clinical, histological, or radiological markers for pituitary aggressive lesions [23–25]. In 2013, Trouillas et al. proposed a classification system for pituitary tumors that could suggest prognostic value for recurrence-free status and tumor progression, comparing anatomical and histologic aspects [10]. In 2015, the same group proposed the role of proliferative markers in classification of pituitary endocrine tumors [26]. Recently, Saeger et al. proposed to provide the proliferative potential and the invasive character separately to define histopathological classification system [22]

In this study, two traditional proliferation markers, p53 and Ki-67, were evaluated, but we also included the analysis of c-erbB2. A strong p53 immunoexpression (positivity ≥ 25% of cells = 3+), present in 60% of the tumors examined, was clearly associated with advanced suprasellar extension (grade 4, according to Edal et al. classification [13]) (*p* = 0.013) and adenoma regrowth/recurrence (*p* = 0.0035). These findings are in accordance with those from previous studies [27–30] related to aggressiveness, invasiveness, and local tumor recurrence interval. However, other studies did not find this correlation, which raised doubts whether this is a reliable recurrence marker [31–33]. In addition, its quantification method still needs to be validated [27, 31].

The expression of Ki-67, a cellular proliferation indicator, is usually detected by the MIB-1 monoclonal antibody. It is expressed in terms of percentage of immunoreactive cellular nuclei and has been extensively used as a biomarker in several neuroendocrine neoplasias [34]. However, its use in pituitary tumors is still questionable due to heterogeneity of studies, with lack of technical standardization, and the use of semiquantitative methods [35, 36]. In our series, the Ki-67 cutoff was established when positivity was found in ≥ 10% (2+) of cells, according to local standards [14, 15]. Some authors suggested that high immunostaining of Ki-67 could predict aggressive tumor behavior and distinguish invasive from noninvasive adenomas with high sensitivity and specificity [35]. Other studies did not find significant association between this biomarker expression and tumor invasion or extension [36–38].

In this study, younger patients presented stronger immunostaining for p53 (>3+) and Ki-67 (>2+) (*p* = 0.021 and *p* < 0.001, resp.). Some of these patients were in isolated familial pituitary adenoma (FIPA) setting. Several studies have described the aggressive behavior of pituitary adenomas in younger ages that might be related to genetic syndromes, as MEN-1, AIP mutations, or X-Lag Syndrome [39–41].

The protooncogene c-erbB2 is a codifier of the epidermal growth factor receptor family. When there is any unusual expression or receptor mutation, migration and invasion patterns emerge, and an evasion of the apoptotic process leads to prolonged cell survival [42–44]. A study previously published by the authors [14] showed that c-erbB2 was expressed in 79% of aggressive nonfunctioning adenomas and in 52% of GH or prolactin secreting adenomas [15].

In this series, c-erbB2 receptor expression is observed in 67% of all aggressive pituitary tumors. However, no independent association was found between c-erbB2 expression and invasion/extension parameters or tumor behavior within the follow-up period considered in this study.

The results revealed that parasellar invasion was significantly related with tumor regrowth/recurrence (*p* = 0.0389). Adenomas with infrasellar invasion required multimodal treatment (*p* = 0.0219), which is a possible indicator of difficulties in surgical management. In particular, in our series, tumors having major contact with the third ventricle and the adjacent cerebral parenchyma may be classified as invasive. Some studies have suggested that the main prognostic factors for prediction of free-disease status [10] are the potential of massive surgical removal [44–47]; the presence of pituitary tumor invasion indicates recurrence/progression [47, 48].

In this study, pituitary tumors were classified based on a combination of invasion parameters and proliferative markers in order to evaluate prognostic impact and tumor behavior. Classically, pituitary tumor invasion is defined by radiological and/or histological involvement of para- and infrasellar regions [12, 13, 47, 48]. Our results suggest that presence of advanced suprasellar extension, Edal et al. 4 [13], with third ventricle or adjacent cerebral parenchyma compression, causing obstructive hydrocephalus, should be considered as an additional invasion parameter.

Even though p53 expression was independently associated with tumor behavior, it is suggested that an immunohistochemical diagnostic evaluation be conducted with more than one biomarker in order to improve sensitivity in predicting pituitary tumors prognosis. Therefore, proliferative tumors, but mainly those classified as grade 2B (invasive-proliferative), showed a significant relation with tumor regrowth/recurrence rate (*p* = 0.0127), confirming that these lesions must be considered as highly suspicious of neoplastic proliferation [10].

Considering the GIS-aided mapping, the authors of this study have recently published and validated for the first time the use of this method to evaluate geographical patterns of acromegaly distribution in the central region of Brazil [16]. The Federal District of Brazil is an administrative area, with a low concentration of dangerous environmental hazardous sources, considering that there are no major industries in this region. Most patients live in urban areas, which include mostly small apartment buildings and houses, and none of them previously worked in industries or large farms. Hence, the population described in this study was homogeneously urban, with comparable sociocultural conditions. In this study, environmental factors that could impact tumor behavior, regrowth/recurrence, or free-disease status during long-term follow-up were not identified. Also, no unexpected clusters were found, and reasonable access to medical care was evidenced. There was no observed association between geographical data and tumor regrowth throughout the study period.

## 7. Conclusion

To our knowledge, this is the retrospective study with the longest median postoperative follow-up ever published that evaluated invasion, proliferation, and epidemiogeographical parameters correlated with pituitary tumor behavior. Ki-67 still presents discrepancies, and c-erbB2 apparently has no influence as a predictor proliferation marker on pituitary tumors. Even if p53 expression was solely independently associated with tumor behavior, it is suggested that an immunohistochemical diagnostic evaluation be conducted with more than one biomarker in order to gain sensitivity in predicting pituitary tumor prognosis. Signs of parasellar and infrasellar invasion remain strong predictors of tumor recurrence and are indicative of the need for multimodal treatment, being thus difficult to manage. However, advanced suprasellar extension with third ventricle or adjacent cerebral parenchyma compression should be considered an invasion parameter, even by its marked association with p53 expression. Even though we could not establish a conclusive link between pituitary tumor behavior and local environmental features, these results may provide future guidance for the comprehension of the environmental aspects that could affect the pathogenesis of pituitary tumors. GIS (geographic information systems) mapping is a useful tool to identify health diagnosis patterns and to regionally improve medical assistance networks.

## Figures and Tables

**Table 1:** Used antibodies, clones, and dilutions.

Antibodies (all Dako Corporations products)	Clones	Dilution
Anti-PRL	Polyclonal	1 : 2000
Anti-GH	Polyclonal	1 : 2000
Anti-FSH	C10	1 : 50
Anti-LH	C93	1 : 50
Anti-ACTH	O2A3	1 : 100
Anti-TSH	42	1 : 50
p53	DO7	1 : 100
Ki-67	MIB 1	1 : 100
c-erbB2	Oncoprotein C	1 : 400

**Table 2:** Radiological and immunohistochemical characterization of tumors on diagnosis.

Radiological aspects	Total (%)
	*n* = 50
Tumor size (mm)	44.7 ± 13.6
Parasellar invasion	42 (84)
Infrasellar invasion	40 (80)
Suprasellar extension	49 (98)
3rd ventricle extension	32 (64)

Immunohistochemistry	Total (%)

*Hormonal expression*	
GH	14 (28)
PRL	8 (16)
ACTH	3 (6)
LH/FSH	7 (14)
*Null cell*	18 (36)
*p53*	
≥3+	29 (60)
*Ki-67*	
≥2+	16 (33)
*c-erbB2*	
≥2+	31 (67)

Clinicopathological classification	Total (%)

Grades	
1A	2 (4)
1B	1 (2)
2A	21 (42)
2B	26 (52)

Values expressed as mean ± SD or frequency (%).

**Table 3:** Evaluation of p53, Ki-67, and c-erbB2 tumor proliferation markers based on clinical and radiological parameters.

	p53		*p* ^*∗*^	Ki-67		*p* ^*∗*^	c-erbB2		*p* ^*∗*^
	<3+	≥3+		<2+	≥2+		<2+	≥2+	
*Age (years)* ^†^	42 ± 15	31 ± 16	0.021	42 ± 15	23 ± 12	<0.001	35 ± 16	35 ± 17	0.955
*Diameter (mm)* ^†^	40 ± 10	50 ± 10	0.062	40 ± 10	50 ± 10	0.009	50 ± 10	40 ± 10	0.937
*Invasion*									
Parasellar			0.235			0.701			0.703
No	5 (26)	3 (10)		6 (19)	2 (13)		2 (13)	6 (19)	
Yes	14 (74)	26 (90)		26 (81)	14 (87)		13 (87)	25 (81)	
Infrasellar			0.451			0.238			0.242
No	5 (26)	4 (14)		8 (25)	1 (6)		1 (7)	7 (23)	
Yes	14 (74)	25 (86)		24 (75)	15 (94)		14 (93)	24 (77)	
*Extension *									
Suprasellar			0.395			1.000			1.000
No	1 (5)	0 (0)		1 (3)	0 (0)		0 (0)	1 (3)	
Yes	18 (95)	29 (100)		31 (97)	16 (100)		15 (100)	30 (97)	
3rd ventricle			0.013			0.087			0.421
No	11 (58)	6 (21)		14 (44)	3 (19)		4 (36)	12 (39)	
Yes	8 (42)	23 (79)		18 (56)	13 (81)		11 (64)	19 (61)	

^*∗*^ *p* value computed using Student's *t*-, Pearson's chi-square, or Fisher's exact test.

^†^Mean ± standard deviation or frequency (%).

**Table 4:** Clinical and radiological features and therapeutic intervention.

Age and radiological aspects	Therapeutic interventions^*∗*^				*p* value
	Only surgery (%)	Surgery and medical treatment (%)	Surgery and radiotherapy (%)	Surgery, medical treatment, and radiotherapy (%)	
*Patients n* (%)	11 (22)	20 (40)	11 (22)	8 (16)	

*Age (years)* ^†^	40 ± 13	29 ± 16	48 ± 11	23 ± 15	0.0008
*Diameter (mm)*	43.7 ± 17.5	46.3 ± 12.2	44.7 ± 15.3	41.9 ± 9.8	0.8812
*Invasion *					
Parasellar					0.7288
No	1 (13)	3 (37)	3 (37)	1 (13)	
Yes	10 (24)	17 (40)	8 (19)	7 (17)	
Infrasellar					0.0219
No	5 (50) [2]	1 (10) [−2]	1 (10) [−1]	3 (30) [1]	
Yes	6 (15) [−2]	19 (48) [2]	10 (25) [1]	5 (12) [−1]	
*Extension *					
Suprasellar					0.1600
No	0 (0)	0 (0)	0 (0)	1 (100)	
Yes	11 (22)	20 (41)	11 (22)	7 (14)	
3rd ventricle					0.1805
No	5 (28)	4 (22)	4 (22)	5 (28)	
Yes	6 (19)	16 (50)	7 (22)	3 (9)	

^*∗*^Values expressed as mean ± standard deviation or frequency (%) and [Pearson residual].

^†^Tukey test results: surgery + radiotherapy versus surgery + medical treatment (*p* = 0.0040) and surgery + radiotherapy versus surgery + radiotherapy + medical treatment (*p* = 0.0022).

*p* value computed using Pearson's chi-square test.

**Table 5:** Immunohistochemical profile, tumor classification, and therapeutic intervention.

	Therapeutic interventions^*∗*^				*p* value
	Only surgery (%)	Surgery and medical treatment (%)	Surgery and radiotherapy (%)	Surgery, medical treatment, and radiotherapy (%)	
*Patients n* (%)	11 (22)	20 (40)	11 (22)	8 (16)	

*Immunohistochemistry*					
*Hormone expression*					0.0186
GH (*n* = 14)	1 (7) [−2]	9 (64) [2]	0 (0.00) [−2]	4 (29) [1]	
PRL (*n* = 8)	1 (13) [−1]	6 (74) [2]	0 (0.00) [−2]	1 (13) [0]	
ACTH (*n* = 3)	1 (33) [0]	0 (0) [−1]	1 (33) [0]	1 (33) [1]	
LH/FSH (*n* = 7)	2 (29) [0]	2 (29) [−1]	3 (42) [1]	0 (0) [−1]	
*Null cell* (*n* = 18)	6 (33) [1]	3 (17) [−2]	7 (39) [2]	2 (11) [−1]	
*Tumor markers*					
*p53*					0.1527
<3+	4 (21)	4 (21)	7 (37)	4 (21)	
≥3+	7 (24)	14 (48)	4 (14)	4 (14)	
*Ki-67*					0.0110
<2+	10 (31) [2]	8 (25) [−2]	10 (31) [2]	5 (13) [−1]	
≥2+	1 (6) [−2]	10 (63) [2]	1 (6) [−2]	4 (25) [1]	
*c-erbB2*					0.3018
<2+	4 (27)	8 (53)	2 (13)	1 (7)	
≥2+	7 (23)	9 (29)	9 (29)	6 (19)	

*Clinicopathological classification*					0.6858
1A	1 (50)	0 (0)	0 (0)	1 (50)	
1B	0 (0)	0 (0)	1 (100)	0 (0)	
2A	4 (19)	9 (43)	5 (24)	3 (14)	
2B	6 (23)	11 (43)	5 (19)	4 (15)	

^*∗*^Values expressed as frequency (%) and [Pearson residual].

*p* value computed using Pearson's chi-square test.

**Table 6:** Clinical and radiological features and tumor evolution classes.

Clinical and radiological aspects at diagnosis	Clinical outcomes			*p* value^*∗*^
	Regrowth/recurrence (%) (*n* = 17)	Stability (%) (*n* = 13)	Reduction/cure (%) (*n* = 20)	
*Gender*				0.399
Male	6 (35)	8 (62)	10 (50)	
Female	11 (65)	5 (38)	10 (50)	
*Age (years)* ^†^	34 ± 19	40 ± 12	32 ± 17	0.419^‡^
*Diameter (mm)* ^†^	44.5 ± 13.9	37.8 ± 13.2	49.4 ± 12.1	0.052^‡^
<30 mm	0 (0)	2 (15)	0 (0)	0.073
30–40 mm	7 (41)	3 (23)	4 (20)	
>40 mm	10 (59)	8 (62)	16 (80)	
*Invasion*				
Parasellar				0.038
No	1 (6) [−1]	5 (38) [3]	2 (10) [−1]	
Yes	16 (94) [1]	8 (62) [−3]	18 (90) [1]	
Infrasellar				0.552
No	5 (29)	2 (15)	3 (15)	
Yes	12 (71)	11 (85)	17 (85)	
*Extension*				
Suprasellar				0.2600
No	0 (0)	1 (8)	0 (0)	
Yes	17 (100)	12 (92)	20 (100)	
3rd ventricle				0.0577
No	6 (35)	8 (62)	4 (20)	
Yes	11 (65)	5 (38)	16 (80)	

^*∗*^Pearson's chi-square test; ^†^values expressed as mean ± standard deviation or frequency (%) and [Pearson residual]; ^‡^ANOVA test.

**Table 7:** Immunohistochemical profile, classification, and tumor evolution classes.

	Clinical outcomes			*p* value^*∗*^
	Regrowth/recurrence (%) (*n* = 17)	Stability (%) (*n* = 13)	Reduction/cure (%) (*n* = 20)	
*Immunohistochemistry*				
*Hormone expression*				0.2193
GH	3 (18)	2 (15)	9 (45)	
PRL	3 (18)	1 (8)	4 (20)	
ACTH	2 (11)	1 (8)	0 (0)	
LH/FSH	3 (18)	1 (8)	3 (15)	
*Null cell*	6 (35)	8 (61)	4 (20)	
*p53*				0.0035
<3+	3 (18) [−2]	10 (77) [3]	6 (33) [−1]	
≥3+	14 (82) [2]	3 (23) [−3]	12 (67) [1]	
*Ki-67*				0.2329
<2+	11 (65)	11 (85)	10 (56)	
≥2+	6 (35)	2 (15)	8 (44)	
*c-erbB2*				0.7118
<2+	4 (25)	5 (38)	6 (35)	
≥2+	12 (75)	8 (62)	11 (65)	

*Classification*				
*Anatomical*				0.5155
Noninvasive	1 (6)	2 (15)	0 (0)	
Invasive	16 (94)	11 (85)	20 (100)	
*Pathological*				0.0145
Nonproliferative	4 (24) [−2]	10 (77) [3]	9 (45) [0]	
Proliferative	13 (76) [2]	3 (23) [−3]	11 (55) [0]	
*Clinicopathological*				0.0127
Grades				
1A	0 (0) [−1]	2 (15) [2]	0 (0) [−1]	
1B	1 (6) [1]	0 (0) [−1]	0 (0) [−1]	
2A	4 (24) [−2]	8 (62) [2]	9 (45) [0]	
2B	12 (70) [2]	3 (23) [−2]	11 (55) [0]	

^*∗*^Pearson's chi-square test; values expressed as frequency (%) and [Pearson residual] were tested to identify variables that contributed to statistical significance.

## Appendix

### A. Antibodies, Clones, and Dilution Protocols Used in This Study

See Table 1.

### B. Immunochemistry: Data Analyses

- GH, PRL, FSH, LH, TSH, and ACTH antibodies (positive cytoplasmic staining):
  1. less than 10% of cells with positive staining,
  2. between 10 and 20% of cells with positive reaction,
  3. between 21 and 50% of cells with positive reaction,
  4. more than 51% of cells with positive reaction.

Note: we considered, as secreting, tumors with any staining intensity.

(B) c-erbB2 (positive: membrane staining):
  (0+) lack of positivity in more than 90% of tumor cells,
  (1+) discrete immunopositivity in more than 10% of tumor cells,
  (2+) discrete to moderate positivity in more than 10% of tumor cells,
  (3+) intense and complete immunoreactivity in more than 10% of tumor cells.
(C) Ki-67 an p53 (positive: nuclear staining):
  (0+) complete lack of positivity in tumor cell,
  (1+) immunoexpression in less than 10% of tumor cells,
  (2+) immunoexpression between 10 and 25% of tumor cells,
  (3+) immunoexpression between 25 and 50% of tumor cells,
  (4+) immunoexpression in more than 50% of tumor cells.
